# Risk stratification biomarkers for *Staphylococcus aureus* bacteraemia

**DOI:** 10.1002/cti2.1110

**Published:** 2020-02-13

**Authors:** Yi Cao, Alessander O Guimaraes, Melicent C Peck, Oleg Mayba, Felicia Ruffin, Kyu Hong, Montserrat Carrasco‐Triguero, Vance G Fowler, Stacey A Maskarinec, Carrie M Rosenberger

**Affiliations:** ^1^ Bioinformatics and Computational Biology Genentech, Inc. South San Francisco CA USA; ^2^ Biomarker Discovery Genentech, Inc. South San Francisco CA USA; ^3^ Clinical Sciences Genentech, Inc. South San Francisco CA USA; ^4^ Division of Infectious Diseases Duke University Durham NC USA; ^5^ BioAnalytical Sciences Genentech, Inc. South San Francisco CA USA; ^6^ BioAnalysis, Immune‐Onc Therapeutics Palo Alto CA USA

**Keywords:** bacteraemia, endocarditis, prognostic biomarkers, *Staphylococcus aureus*

## Abstract

**Objectives:**

To identify risk stratification biomarkers to enrich for the subset of *Staphylococcus aureus* bacteraemia patients who develop deep‐seated tissue infections with high morbidity and mortality to guide clinical trial enrolment and clinical management.

**Methods:**

We evaluated the prognostic value of eight biomarkers for persistent bacteraemia, mortality and endovascular infection foci in a validation cohort of 160 patients with *S. aureus* bacteraemia enrolled consecutively over 3 years.

**Results:**

High levels of IL‐17A, IL‐10 or soluble E‐selectin at bacteraemia diagnosis correlated with the duration of positive blood cultures. When thresholds defined in an independent cohort were applied, these biomarkers were robust predictors of persistent bacteraemia or endovascular infection. High serum levels of IL‐17A and IL‐10 often preceded the radiographic diagnosis of infective endocarditis, suggesting potential utility for prioritising diagnostic radiographic imaging. High IL‐8 was prognostic for all‐cause mortality, while IL‐17A and IL‐10 were superior to clinical metrics in discriminating between attributable mortality and non‐attributable mortality. High IL‐17A and IL‐10 identified more patients who developed microbiological failure or mortality than were identified by infective endocarditis diagnosis.

**Conclusion:**

These biomarkers offer potential utility to identify patients at risk of persistent bacteraemia to guide diagnostic imaging and clinical management. Low biomarker levels could be used to rule out the need for more invasive TEE imaging in patients at lower risk of infective endocarditis. These biomarkers could enable clinical trials by enriching for patients with the greatest need for novel therapies.

## Graphical abstract

We found that serum IL‐17A, IL‐10 and sE‐selectin are prognostic for persistent bacteraemia and infective endocarditis in patients hospitalised with *S. aureus* bacteraemia. High serum levels often preceded the radiographic diagnosis of infective endocarditis, suggesting potential utility for risk stratification. High IL‐17A identified more patients who meet clinical study endpoints of antibiotic failure than were identified by infective endocarditis diagnosis, offering potential utility as risk stratification biomarkers to facilitate patient selection for clinical trials.

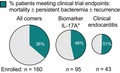

## Introduction

Mortality rates for patients with *Staphylococcus aureus* bacteraemia (SAB) largely remain unchanged despite significant advances in clinical management, with new antibiotic therapies and rapid diagnostics available in recent decades. Significant heterogeneity in the clinical course of patients with SAB despite appropriate antibiotic therapy suggests that differential host immune response may contribute to adverse patient outcomes.^1^, ^2^, ^3^ However, critical determinants of immune evasion in patients with SAB remain poorly understood and connections between the immune response and microbiological failure are lacking. Previous studies identified several candidate innate immunity biomarkers that were independently associated with mortality and prolonged SAB, supporting the utility of biomarkers to distinguish patients prone to developing severe disease.^1^, ^4^, ^5^, ^6^, ^7^ Such information would be particularly beneficial in guiding diagnostic work‐up of patients at increased risk of *S. aureus* infective endocarditis (IE), a disease that even with the best available treatments has contemporary in‐hospital mortality rates ranging from 25 to 46%.^8^, ^9^, ^10^

To investigate the contribution of the differential immune response to SAB disease severity, we previously identified biomarker profiles associated with mortality and persistent bacteraemia in a two‐phase case–control study of patients with complicated SAB. There, we evaluated a panel of 13 candidate biomarkers in patients with complicated SAB enriched for persistent bacteraemia and mortality and observed that high IL‐17A, IL‐10 and soluble E‐selectin (sE‐selectin) levels at presentation were prognostic for persistent bacteraemia, while high levels of IL‐8 and CCL2 were prognostic for mortality.^1^ In the validation cohort presented herein, we evaluated the top 8 biomarkers with the strongest prognostic value in an unselected population of 160 patients with SAB consecutively enrolled at a single centre over a three‐year period. Consistent findings across these two studies demonstrate that heterogeneity in host biomarker levels present at SAB diagnosis could be informative to identify and direct the clinical management of patients at greatest risk of poor outcomes. These biomarkers also provide potential risk stratification tools for interventional trials to enrich for patients with SAB most likely to fail standard‐of‐care antibiotics.

## Results

### Validation of prognostic biomarkers for persistent bacteraemia in an unselected cohort

We previously identified a panel of eight biomarkers (IL‐17A, IL‐10, sE‐selectin, IL‐6, Angpt2, IL‐1RN, IL‐8 and CCL2) with the strongest prognostic value for persistent bacteraemia and mortality in a cohort of patients with complicated SAB enriched for these outcomes.^1^ In this study, we evaluated this biomarker panel in an unselected validation cohort of 160 patients with SAB consecutively enrolled over three years. Cohort characteristics are provided in Table 1.

**Table 1:** Cohort characteristics

Number of patients	160
Age (years), median (range)	61 (21–94)
Female, no. (%)	66 (41)
Outcome, no (%)	
Mortality, 30 days	15 (9)
Attributable mortality	6 (4)
Mortality, 30 days	9 (6)
In‐hospital mortality	7 (4)
Persistent bacteraemia	44 (28)
Complicated infection	160 (100)
Recurrence	8 (5)
Hospital LOS (days), median (range)	14 (4–109)
Treatment duration (days), median (range)	45 (7–190)
APACHE II score	15 (0–49)
MRSA, no. (%)	67 (42)
Cardiac device present, no. (%)	26 (16)
Infection foci, no. (%)	
Endovascular	80 (50)
Infectious endocarditis (IE)	43 (27)
Non‐IE	37 (23)
Extravascular	80 (50)
Osteoarticular	35 (22)
Soft tissue	18 (11)
Other	27 (17)
Comorbidities, no. (%)	
Diabetes	70 (44)
Haemodialysis dependent	16 (10)
Cancer	27 (17)
Transplant recipient	8 (5)
HIV+	2 (1)

Serum was collected 1–3 days after positive blood culture for *S. aureus* bacteraemia. Patients with more than one identified focus of infection were assigned to multiple infection focus categories in this table. Other foci include central venous catheter‐associated infections and unidentified sources of infection.

APACHE II, Acute Physiologic Assessment and Chronic Health Evaluation score; LOS, hospital length of stay; MRSA, methicillin‐resistant *S. aureus*.

**Figure 1:**
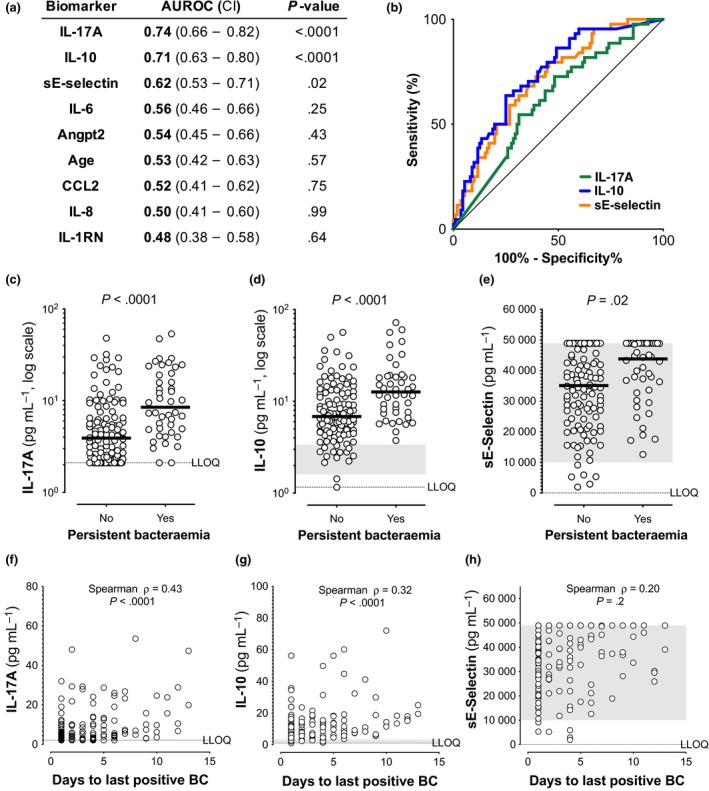
Biomarkers prognostic for persistent bacteraemia. **(a)** Baseline biomarker area under the receiver operating characteristic curve with 95% confidence intervals [AUROC (CI)] and unadjusted Mann–Whitney *U*‐test *P*‐values between persistent and non‐persistent bacteraemia. **(b)** ROC curves for biomarkers significantly prognostic for persistence identified in a (*P* < 0.05). **(c–e)** Significantly higher IL‐17A, IL‐10 and sE‐selectin levels at presentation in patients with persistent vs. non‐persistent bacteraemia. Median values as black bars, *P* = unadjusted Mann–Whitney *U*‐test. **(f–h)** Spearman correlations (rho and *P*‐values) between duration of positive blood culture (BC) and higher IL‐17A, IL‐10 or sE‐selectin. *n* = 156 subjects with available blood culture data. Dotted lines indicate lower limit of quantification (LLOQ) (2.1 pg mL^−1^ for IL‐17A, 1.2 pg mL^−1^ for IL‐10 and 5.1 pg mL^−1^ for sE‐selectin) or upper limit of quantification (ULOQ) (48 920 pg mL^−1^ for sE‐selectin). Grey shaded area represents the range of the healthy control values for IL‐10 and sE‐selectin, with healthy IL‐17A levels below LLOQ (*n* = 16). The ULOQ for sE‐selectin (48 920 pg mL^−1^) was assigned for 25% (28/112) of non‐persistent and 34% (15/44) of persistent patients when levels were above this value. Biomarker values shown are the average of technical triplicate measurements.

High IL‐17A, IL‐10 and sE‐selectin levels measured within 1–3 days of the positive index blood culture were prognostic for persistent bacteraemia (defined as positive blood cultures ≥ 5 days from the index blood culture) on appropriate antibiotic therapy (Figure 1a–e and Supplementary table 1). In contrast, known risk factors (age, haemodialysis, corticosteroid use, infection foci (i.e. the presence of endovascular infection), hospital‐ vs. community‐acquired infection, MRSA vs. MSSA), other measured biomarkers and available clinical metrics (APACHE II score, complete blood counts, clinical chemistry) were not prognostic (all *P* > 0.05, data not shown). Higher levels of IL‐17A at presentation showed the best prognostic power for persistent bacteraemia, as indicated by the largest area under the receiver operating characteristic (AUROC = 0.74) curve, followed by IL‐10 (AUROC = 0.71) (Figure 1a, b and Supplementary figure 1). Higher IL‐17A also correlated with longer duration of positive blood cultures better than other biomarkers (Figure 1f, ρ = 0.43, *P* < 0.0001), followed by IL‐10 (Figure 1g, ρ = 0.32, *P* < 0.0001). sE‐selectin was elevated in persistent bacteraemia (Figure 1e) but had less discriminatory power and did not correlate well with duration of positive blood cultures (Figure 1h, ρ = 0.20, *P* = 0.2). No biomarkers predicted which patients would experience recurrence of SAB following hospital discharge by univariate analysis or modelling (*P* > 0.05).

### IL‐17A, IL‐10 and sE‐selectin identify patients with endovascular infections

**Figure 2:**
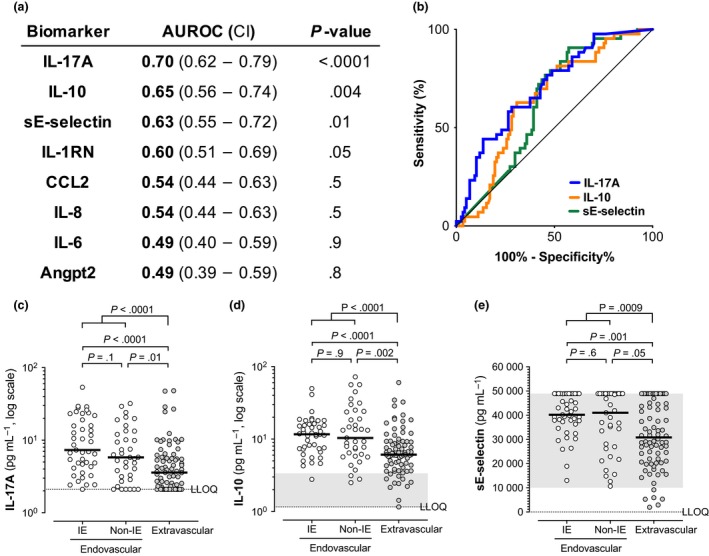
IL‐17A, IL‐10 and sE‐selectin identify patients with infective endocarditis and endovascular infections. **(a)** Baseline biomarker area under the receiver operating characteristic curve with 95% confidence intervals [AUROC (CI)] and unadjusted Mann–Whitney *U*‐test *P*‐values between infectious endocarditis (IE) vs. all other foci. **(b)** ROC curves for biomarkers associated with IE identified in a (*P* < 0.05). **(c–e)** Patients with endovascular infection (white circles) have higher median IL‐17A, IL‐10 and sE‐selectin levels (black bars) than patients with extravascular infections (grey circles). IE, *n* = 43; non‐endocarditis endovascular (non‐IE), *n* = 37; and extravascular (osteoarticular, soft tissue and other foci) infection foci, *n* = 80. Unadjusted Mann–Whitney *U*‐test *P*‐values (*P*), LLOQs and healthy control range as described in Figure 1. Biomarker values shown are the average of technical triplicate measurements.

We evaluated the clinical metrics of subjects presenting with high IL‐17A, IL‐10 or sE‐selectin to understand features in addition to persistent bacteraemia that associate with high levels of these three biomarkers. We observed that higher IL‐17A, IL‐10 and sE‐selectin levels identify patients with IE infection foci (diagnosed by TEE or TTE imaging) when compared to extravascular infections (Figure 2a, b and Supplementary figure 2a–c), while the other five biomarkers, available risk factors or clinical data did not (*P *> 0.05, data not shown). Similarly, high levels of IL‐17A, IL‐10 and sE‐selectin were measured in patients with all endovascular infections (IE + non‐IE) vs. extravascular foci, as well as non‐IE endovascular infections vs. extravascular foci (Figure 2c–e). None of the 8 biomarkers or other clinical metrics evaluated in this study distinguished between IE and non‐IE endovascular infections (Figure 2c–e, data not shown). We previously reported that median IL‐17A was higher in patients with endovascular foci.^1^ Endovascular infections are associated with persistent bacteraemia, and these three biomarkers were also associated with persistent bacteraemia (Figure 1c–e). When patients were grouped by the presence of persistent bacteraemia, we observed higher IL‐17, IL‐10 or sE‐selection in patients with endovascular foci (IE and non‐IE) than extravascular infections only in the non‐persistent group (Supplementary figure 3). Median levels of both biomarkers were higher in patients with persistent bacteraemia regardless of infection foci (Supplementary figure 3). Therefore, high levels of IL‐17A, IL‐10 and sE‐selectin identified both patients at higher risk of persistent bacteraemia and patients with endovascular reservoirs of infection despite surveillance blood cultures rapidly turning negative.

We evaluated whether any combinations of biomarker and clinical data available at presentation, including known clinical risk factors for SAB severity, gave superior prognostic power than individual biomarkers using lasso and random forest modelling. IL‐17A was the only significant predictor identified by lasso modelling and was also the largest contributor in random forest modelling, when predicting either the presence of IE or any endovascular infection (Supplementary figure 2d, e). IL‐17A and other biomarkers weighted in the random forest model (IL‐10, previously reported to be elevated in IE,^5^, ^7^ sE‐selectin and IL‐1RN) were intercorrelated (Supplementary figure 2f, Spearman ρ > 0.4).

### High biomarker levels precede diagnosis of infective endocarditis

**Figure 3:**
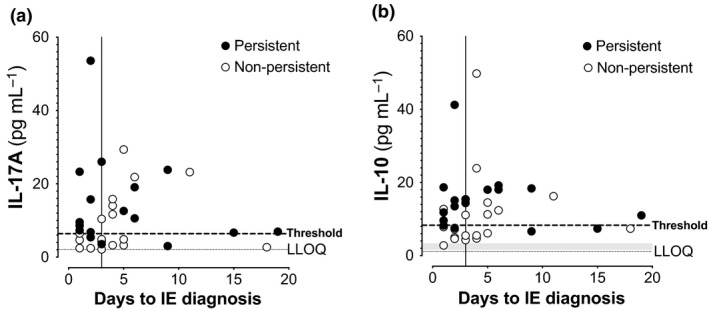
High biomarker levels precede infective endocarditis diagnosis. Serum levels of **(a)** IL‐17A or **(b)** IL‐10 measured within the first 3 days (indicated by vertical line) were plotted against the day of endocarditis diagnosis. Horizontal dotted line indicates biomarker threshold for classifying IE defined using previous cohort to maximise sensitivity and specificity. Thresholds for IL‐17A and IL‐10 identified 71% and 65%, respectively, of IE patients diagnosed after day 3 (to the right of the vertical line). Black circles denote persistent bacteraemia, *n* = 34, with available timing of TEE imaging. LLOQs and healthy control range as described in Figure 1. Biomarker values shown are the average of technical triplicate measurements.

The ability to identify patients with IE at admission could direct their clinical work‐up, including diagnostic imaging. Higher levels of IL‐17A and IL‐10 preceded the diagnosis of IE [first positive transesophageal echocardiography (TEE) or transthoracic echocardiography (TTE)] by several days to weeks in some patients (Figure 3), previously observed for IL‐17A.^1^ Thresholds for IL‐17A and IL‐10 identified 71% and 65%, respectively, of IE patients diagnosed after day 3, the time by when samples were available for biomarker measurements (Figure 3). While thresholds with good sensitivity could be identified, they showed poor specificity (Supplementary table 1). Importantly, while persistent bacteraemia despite effective antibiotic treatment raises the clinical suspicion of IE, these biomarkers also identified patients who developed IE in the absence of persistent bacteraemia (open circles, Figure 3), providing rationale to consider earlier diagnostic imaging and continued surveillance for IE‐related complications.

### IL‐8 is the strongest prognostic biomarker for mortality in the serum biomarker panel

**Figure 4:**
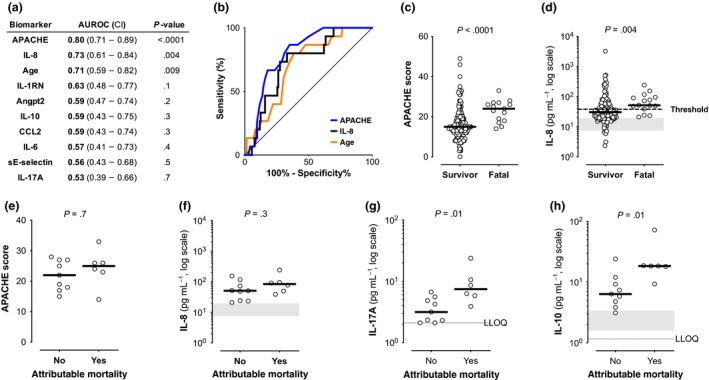
Biomarkers prognostic for mortality. **(a)** Biomarker area under the receiver operating characteristic curve with 95% confidence intervals [AUROC (CI)] and *P*‐values between all‐cause 90‐day mortality vs. survival for baseline serum proteins levels, age and APACHE II score (the only clinical parameters at presentation that were significantly different between fatal cases and survivors), *n* = 160. **(b)** ROC curves comparing prognostic power for all‐cause 90‐day mortality for significant metrics identified in a. **(c, d)** Increased baseline median APACHE II score and serum IL‐8 in fatal cases vs. survivors. Dotted line indicates 38 pg mL^−1^ IL‐8 threshold. **(e–h)** Median APACHE II score or IL‐8 levels did not distinguish between attributable vs. non‐attributable mortality, while IL‐17A and IL‐10 levels were significantly higher in cases of attributable mortality. *n* = 15 fatal cases; unadjusted Mann–Whitney *U*‐test *P*‐values (*P*), LLOQs and healthy control range as described in Figure 1. Biomarker values shown are the average of technical triplicate measurements.

The prognostic value of the serum biomarkers we previously identified as prognostic for mortality (IL‐8, CCL2, IL‐6, IL‐1RN, Angpt2 and IL‐10^1^) was evaluated in this unselected SAB cohort and compared with clinical risk factors (such as age, haemodialysis, corticosteroid use, endovascular infection source, MRSA vs. MSSA, persistent bacteraemia and APACHE II^11^, ^12^) and routine clinical data and laboratories including blood cell counts. Validating our previous observation that IL‐8 had the strongest prognostic value for 90‐day all‐cause mortality among candidate serum biomarkers,^1^ IL‐8, APACHE II score and advanced age were the best predictors of mortality in this cohort, followed by IL‐1RN and Angpt2 (Figure 4a–d). These five variables were also the strongest predictors of 30‐day mortality and in‐hospital mortality with similar AUROC (Supplementary figure 5). The total APACHE II score was more predictive for each mortality outcome than age (Figure 4 and Supplementary figure 5). In evaluating the components of APACHE II, the acute physiology score and chronic health points were significantly higher for 30‐day and 90‐day mortality, while age points (age ranges assessed as categories rather than a continuous variable) were not [acute physiology score (Supplementary figure 4h): AUROC – 90‐day mortality: 0.69, 30‐day mortality: 0.66 and in‐hospital mortality: 0.70; chronic health points: chi‐squared test < 0.05; and age points: chi‐squared test > 0.05]. 80 pg mL^−1^ IL‐8 had been identified in the discovery cohort as the threshold with the highest combined specificity and sensitivity but classified only 33% of patients correctly when applied to the validation cohort (Figure 4c and Supplementary table 1). However, a threshold of 38 pg mL^−1^ IL‐8, calculated to maximise sensitivity and specificity in this cohort, correctly classified fatal outcomes with ≥ 80% sensitivity (Figure 4d and Supplementary table 1). Combinations of biomarkers and clinical comorbidity risk factors were not superior to IL‐8 using a linear regression model (data not shown). Neither IL‐8 nor APACHE II score discriminated between attributable and non‐attributable mortality, consistent with the discovery cohort (Figure 4e, f). Median IL‐17A and IL‐10 levels were significantly higher in attributable mortality vs. non‐attributable mortality (Figure 4g, h). When biomarker associations with all available mortality outcomes were examined in the subgroup of patients with persistent bacteraemia, the only significant associations were for IL‐8 and IL‐1RN for 30‐day mortality (Supplementary figure 6b, c). For the subgroup of patients with non‐persistent bacteraemia, IL‐8 was the biomarker most strongly associated with 30‐day and 90‐day all‐cause and attributable mortality.

### Biomarker‐guided patient selection to enrich for clinical failure outcomes

**Figure 5:**
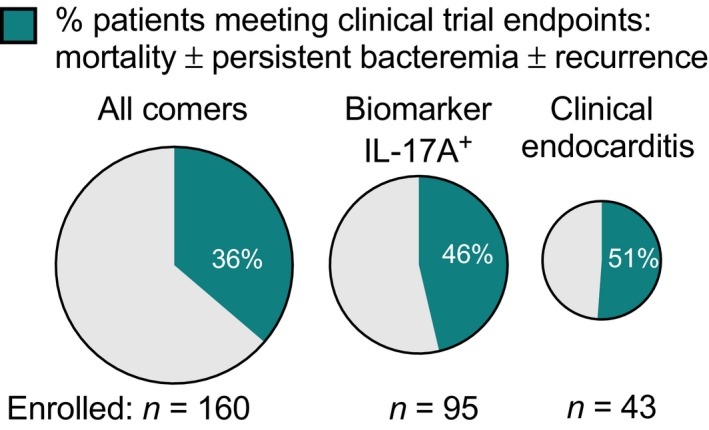
Biomarker‐guided patient selection enriches for potential clinical trial endpoints. This is a graphical representation of Table 2 data, illustrating patient number and frequency of meeting a composite endpoint of mortality ± persistent bacteraemia ± recurrence of bacteraemia within 90 days. The event rate was increased when patients were selected by IL‐17A biomarker‐high patients (≥ 4 pg mL^−1^) or by infective endocarditis diagnosis. Biomarker‐guided enrichment selected twice as many subjects as enriched by clinical IE diagnosis, increasing the feasibility of clinical trial enrolment.

We assessed the ability of biomarkers to identify patients with all‐cause or attributable mortality and microbiological failure (persistent bacteraemia, recurrence) at day 90 after the diagnosis of SAB. Performance characteristics (sensitivity, specific, positive and negative predictive values) are provided in Supplementary table 1. When thresholds defined in the previously published cohort with maximal sensitivity and specificity for persistent bacteraemia^1^ were applied to this cohort, high IL‐17A increased the incidence of this composite endpoint from 36% to 46% in a 41% smaller population (95 of 160 subjects; Figure 5 and Table 2). Patient selection based on high IL‐10 would increase the incidence to 53% but with a higher screen fail rate (62% smaller population, 60 of 160 subjects). IL‐10 showed comparable performance to IL‐17A if a lower threshold were applied to select the same number of subjects (*n* = 95, data not shown). There was a minimal additive value of combining high IL‐17A and IL‐10 to increase the incidence of mortality or microbiological failure (data not shown), and these biomarkers were well correlated with each other (Spearman ρ = 0.66; Supplementary figure 2f). Because of the low mortality rate, the increased composite endpoint event rate was driven by enrichment of persistent bacteraemia, with high IL‐17A capturing 86% of persistent bacteraemia cases, and increasing the incidence from 28% to 40%, with high IL‐10 increasing the incidence to 45% (Figure 5 and Table 2). Selecting on high IL‐17A included 83% of attributable mortality cases and 60% of all‐cause mortality. High IL‐8 alone or in combination with IL‐17A or IL‐10 did not enrich for mortality better than IL‐17A or IL‐10 alone (data not shown). Selecting IE patients increased the event rate to 51%, similar to biomarker‐guided selection, but excluded 73% of the population, thereby missing 55% of the persistent bacteraemia cases and 62% of the patients failing the composite endpoint (Figure 5 and Table 2). Therefore, biomarker‐guided patient selection provides a potential enrichment strategy to identify a larger number of patients who develop persistent bacteraemia or fatal outcome than selection based on IE.

**Table 2:** Enrichment for severe outcomes using risk stratification biomarkers

	*n* (%) of all comers	Proportion of event captured by biomarker‐high group				Event rate			
		All‐cause mortality	Attributable mortality	Persistence	Mortality–persistence–recurrence	All‐cause mortality	Attributable mortality	Persistence	Mortality–persistence–recurrence
All comers	160 (100%)	15	6	44	58	9%	4%	28%	36%
IL‐17A+	95 (59%)	9 (60%)	5 (83%)	38 (86%)	44 (76%)	9%	5%	40%	46%
IL‐10+	60 (38%)	7 (47%)	5 (83%)	27 (61%)	32 (55%)	12%	8%	45%	53%
IE	43 (27%)	4 (27%)	3 (50%)	20 (45%)	22 (38%)	9%	7%	47%	51%

The proportion of clinical failure events in the entire all‐comers population (*n* = 160) captured by biomarkers or diagnosis of infective endocarditis (IE) are shown on the left. *n* = the number of patients in the biomarker‐high group. The overall event rates for each group are shown on the right. Biomarker thresholds defined in a separate cohort for enriching for persistent bacteraemia were applied to the current study: IL‐17A ≥ 4 pg mL^−1^ and IL‐10 ≥ 11 pg mL^−1^. Similar results were obtained if thresholds defined for each outcome were applied (data not shown). Persistence = blood culture positive for ≥ 5 days. Mortality + Persistence + Recurrence = 90‐day all‐cause mortality and/or persistent bacteraemia and/or recurrent SAB within 90 days.

## Discussion

In this study, the prognostic values of IL‐17A, IL‐10, sE‐selectin and IL‐8 identified in a discovery cohort enriched for microbiological failure and mortality were validated in an unselected cohort of serially enrolled patients with SAB, with very similar AUROC between the two studies. Importantly, high IL‐17A, IL‐10 and sE‐selectin were superior to clinical metrics in risk‐stratifying patients for persistent bacteraemia when using the threshold of 5 days of positive blood cultures, a time frame supported by recent studies.^13^, ^14^ These three biomarkers were correlated with each other, providing a possible explanation for why inclusion of additional biomarkers did not significantly improve the linear model or lasso model. They were also more strongly associated with IE/endovascular infections when compared to clinical risk factors, routinely available laboratory measures and the other five biomarkers. This is consistent with endovascular infection being a risk factor for persistent bacteraemia. Persistent bacteraemia increases mortality risk,^5^, ^6^, ^13^ and high IL‐17A and IL‐10 levels were associated with attributable mortality. We did not confirm the modest correlation between sE‐selectin and duration of positive blood cultures in our previous study (ρ = 0.30, *P* = 0.0007^1^), which could be as a result of a technical limitation in this study resulting in 26% of patients assigned the upper limit of quantification value for sE‐selectin.

The current data support the potential for using biomarkers as a stratification tool for interventional trials in patients with SAB. Smaller trials of new anti‐infectives would be made possible by the ability to select biomarker‐high subjects at greater risk of approvable endpoints (such as mortality, persistent bacteraemia and recurrence). Applying previously defined biomarker thresholds to the current cohort showed that high IL‐17A identified more than twice as many subjects meeting any of the three endpoints compared with selection based on radiographic IE diagnosis. Either IL‐17A or IL‐10 captured > 80% of persistent bacteraemia or attributable mortality with nearly half the screen fail rate (41% for IL‐17A vs. 73% for IE). Additionally, early biomarker levels within the first few days could be used for patient selection at a time when IE was not yet diagnosed in all patients. Rose *et al*. identified IL‐10 is a potential biomarker to identify SAB patients at higher risk of severe clinical outcomes when measured prior to empiric antibiotic therapy.^5^, ^7^, ^15^ We show here that IL‐10 retains prognostic value when measured up to 3 days after initiation of effective antibiotic therapy, consistent with observations in an independent cohort.^6^ The unmet need is in patients with complicated SAB, who are most likely to fail standard‐of‐care antibiotics. Clinical selection criteria and enrichment biomarker measurements available within the first few days would best facilitate clinical trial enrolment. The majority of patients (86%) in this unselected cohort met the criteria of complicated bacteraemia within the first 3 days of presentation, supporting the feasibility of biomarker‐directed patient selection for clinical studies of novel therapeutics.

High levels of IL‐10, IL‐17A and sE‐selectin within the first 3 days of SAB identified patients with IE infection foci and patients at higher risk of persistent bacteraemia, which could guide clinical management. In this unselected cohort of patients enrolled serially over three years, all patients met the IDSA criteria for complicated infection. The prevalence of complicated SAB is increasing, driven in part by increasing numbers of implanted devices, which can be found in more than half of patients with SAB and is one of the criteria for complicated bacteraemia.^16^ Patients with complicated SAB could most benefit from adjunctive biomarker‐guided clinical management and enrolment in clinical trials, and the identified biomarkers may not apply to lower‐risk uncomplicated SAB. Higher‐risk patients could be treated more aggressively with better antibiotic regimens. A proof‐of‐concept study of this approach demonstrated significant mortality reduction in patients treated with daptomycin plus ceftaroline compared with vancomycin alone in the subgroup of patients with elevated IL‐10.^14^

IL‐17A and IL‐10 have potential clinical utility for ruling out the need for more invasive and higher sensitivity TEE diagnostic imaging in favor of TTE imaging for low‐risk biomarker‐negative patients.^17^ Delays in diagnosing IE by imaging (TTE or TEE) can range from days to weeks and is a predictor of valve destruction and requirement for surgery, and significantly, more embolic events were observed with delays of ≥ 4 days.^18^ High serum levels of IL‐17A and IL‐10 preceded the diagnosis of IE by radiographic imaging in many patients. Since levels of these two biomarkers are also elevated in patients with non‐IE endovascular foci and persistent bacteraemia, their specificity in identifying IE will be reduced if the frequency of non‐IE endovascular foci and persistent bacteraemia is high in a population. The biomarkers consistently showed stronger negative predictive value than positive predictive value at the biomarker thresholds calculated to give the highest combined sensitivity and specificity. These biomarkers could also prioritise continued vigilance in monitoring for adequate source control and metastatic infection, especially in those patients with implanted medical devices.

Our observations are consistent with other reports that high IL‐8 is prognostic for all‐cause mortality in SAB patients,^1^, ^5^, ^6^, ^7^, ^19^ including mortality in a cohort with IE caused by a variety of bacteria.^20^ The prognostic value of IL‐8 for mortality is not specific to SAB, as this has been observed across multiple sepsis cohorts including patients with both Gram‐positive and Gram‐negative bacterial infections.^21^, ^22^ We did not replicate our prior finding that the combination of IL‐8 + CCL2, IL‐10 or other biomarkers in our panel was prognostic for 90‐day mortality.^1^ IL‐10, IL‐8 and IL‐6 have been associated with 30‐day or in‐hospital mortality.^5^, ^6^, ^7^, ^15^ We also evaluated mortality over shorter windows of 30 days or in‐hospital, since comorbidities can be more impactful for the longer 90‐day outcome.^23^ While IL‐8 and IL‐1RN were more strongly associated with all three mortality metrics than IL‐10 in this cohort, the number of fatal outcomes was small (*n* = 15 90‐day, *n* = 9 30‐day mortality and *n* = 7 in‐hospital mortality). While we expected sufficient power to be able to replicate our prior findings based on the prior effect size, we observed higher biomarker levels in attributable vs non‐attributable mortality across both studies (Figure 4, data not shown). Attributable mortality was less frequent in the current cohort (*n* = 6, 40% of all‐cause mortality) than the prior cohort (*n* = 18, 67% of all‐cause mortality), offering a potential explanation for differential biomarker associations across cohorts that should be evaluated in future studies. IL‐10 or IL‐17A did discriminate between attributable mortality and non‐attributable mortality (*P* = 0.01), while in the discovery cohort, IL‐17A was not significantly different between the two causes of mortality (IL‐17A, *P* = 0.14^1^).

Of the previously published cytokines prognostic for persistent bacteraemia, this study validated IL‐10 and IL‐17A.^1^, ^6^ High IL‐10 is associated with higher circulating levels of bacteria.^7^ The validation of IL‐17A is noteworthy given that few biomarkers to aid in the diagnosis or prognosis of IE have been validated across cohorts (reviewed by Snipsøyr *et al.* 24). IL‐17A is genetically associated with *Staphylococcal* infections, and data from skin and acute *S. aureus* infection mouse models support a protective role for IL‐17.^25^, ^26^, ^27^, ^28^, ^29^ While systemic levels of IL‐17A can serve as a biomarker of chronic *S. aureus* tissue infection, antibody‐mediated blockade of IL‐17 1 week after haematogenous seeding of tissues did not indicate a mechanistic role for IL‐17A in driving bacterial persistence in infected foci between 2 and 4 weeks of infection (data not shown). Further mechanistic studies to understand which prognostic biomarkers drive severe clinical outcomes could enable the development of adjunctive therapeutic strategies to modulate the host response to infection.

Host factors have been proposed to play a larger role than pathogen genotype, particularly for longer‐term mortality rate (i.e. 90 days).^23^ Responses can be host‐ or pathogen‐driven, and we have observed that high levels of bacterial DNA in whole blood, plasma or serum were associated with endovascular foci, persistent bacteraemia and mortality.^30^, ^31^ *Staphylococcus aureus* genotypes (i.e. clonal complexes CC30 and CC5) have been associated with complicated infections such as endocarditis and haematogenous complications.^32^, ^33^, ^34^ Bacterial genotype information was not available for enough patients in this study to support analysis of how bacterial virulence may drive severity, but this interesting question merits analysis in larger studies.

Study limitations include enrolment of this retrospective study at a single tertiary referral centre and lack of evaluation of the timeliness and adequacy of source control by independent surgeons.^35^ Important clinical metrics of organ failure (SOFA score) and sepsis (SEPSIS‐3) were not available to evaluate relationships between the biomarker panel and a broader range of disease severity. We observed that serum biomarkers were superior to other available clinical comorbidity risk factors but not APACHE II severity scores or age; while APACHE has been assessed as superior to Pitt bacteraemia score in predicting SAB mortality, the Pitt bacteraemia scores, Charlson comorbidity index scores and the recently described MRSAB risk score were not available for this cohort for comparative analysis.^36^, ^37^, ^38^ Future cohorts with longitudinal sampling would allow evaluation of whether the duration of high levels of the biomarkers associated with endovascular infections could be used to monitor clearance of infection foci in clinical trials. The 90‐day window for assessment of mortality could impair the ability of clinicians to discriminate between attributable mortality and non‐attributable mortality, and mortality may be less strongly connected to initial biomarker levels. Unselected cohorts with greater numbers of in‐hospital or 30‐day mortality than available in the current cohort would support prognostic biomarker associations with these mortality outcomes. Evaluation of biomarkers in a prospective study would assess their value in prioritising more rapid diagnosis and optimal treatment and for patient stratification in clinical trials.

## Methods

### Experimental design

Consecutive samples from subjects with confirmed *S. aureus* bacteraemia between 2014 and 2016 were obtained from a Duke University (Durham, NC, USA) Biobank. All subjects received appropriate antibiotic therapy, including vancomycin and zosyn (piperacillin and tazobactam), upon SAB diagnosis. Samples were collected with informed consent under protocols approved by the Institutional Ethics Review Boards (IRB protocol Pro00008031). Serum samples were collected within 1–3 days of the positive index blood culture. Serum samples from 16 healthy volunteers were obtained through the Genentech Samples for Science programme.

### Clinical severity definitions

The Infectious Disease Society of America definition was used to retrospectively assign complicated bacteraemia.^39^ All‐cause mortality was assessed within 30 days or 90 days or in‐hospital. Infection‐related mortality was assessed by retrospective chart review by two Board‐certified infectious disease physicians using the following criteria: culture positive and/or persistent infection focus and/or systemic signs and symptoms at the time of death or attending physician considered death to be attributable to SAB. Persistent bacteraemia was defined as any positive blood culture on or after 5 days during the initial hospitalisation, regardless of whether there was a preceding negative blood culture.^13^, ^14^ Seven patients classified as persistent bacteraemia had positive blood cultures following negative cultures. Duration of positive blood culture was calculated as days to last positive blood culture. Recurrence was defined as microbiologically confirmed SAB after hospital discharge during the 90‐day follow‐up window. Infectious endocarditis was diagnosed by the presence of vegetation by TTE or TEE radiographic imaging.

### Prognostic metrics

Serum biomarkers were measured using multiplexed microfluidic immunoassay kits (Ella platform; ProteinSimple, San Jose, CA, USA), with reported values, the average of technical triplicate measurements. These were compared with known severity risk factors and other available clinical data: % and absolute blood cellularity (white blood cell count, lymphocytes, neutrophils, bands), platelets, HCT, BUN, creatinine, age, gender, race, infection foci (endovascular, osteoarticular, soft tissue, other), diabetes mellitus, haemodialysis dependence, corticosteroid use, methicillin resistance, hospital‐ vs community‐acquired infections, persistent bacteraemia and APACHE II severity score.

### Statistical analysis

The Mann–Whitney *U*‐test *P*‐values and non‐parametric Spearman rank correlation coefficients are reported. All statistical analyses were performed with the R program^40^ or Prism software (GraphPad Software, San Diego, CA, USA). The discriminatory power of each variable was assessed by the area under the receiver operating characteristic (AUROC) curve on log‐transformed data using the R package pROC.^41^ The optimal biomarker threshold was calculated using data from a previously published cohort^1^ using Youden's index *J* (*J* = sensitivity + specificity − 1 is maximised) and applied to this study. To identify the minimal set of variables in the prognosis of the binary patient outcomes while minimising the possibility of overfitting, all measured variables were treated as covariates in a lasso model (with 10‐fold cross‐validation) using the R package glmnet,^42^ where we evaluated both the deviance and misclassification minimisation approaches. We used random forest^43^ as an alternative approach to identify the main covariates driving patient classification. All biomarkers, available clinical risk factors for SAB severity, clinical metrics, blood chemistry and cellularity were included in modelling analyses.

## Conflicts of Interest

YC, AOG, MCP, OM, KH, MC‐T and CMR were employees of Genentech, Inc. during the execution of this study. VGF served as Chair of V710 Scientific Advisory Committee (Merck); has received grant support from Cerexa/Actavis/Allergan, Pfizer, Advanced Liquid Logics, NIH, MedImmune, Basilea, Karius, ContraFect, Regeneron and Genentech; has NIH STTR/SBIR grants pending with Affinergy, Locus and Medical Surface, Inc; has been a paid consultant for Achaogen, Astellas, Arsanis, Affinergy, Basilea, Bayer, Cerexa, ContraFect, Cubist, Debiopharm, Durata, Grifols, Genentech, MedImmune, Merck, Medicines Co., Pfizer, Novartis, NovaDigm, Theravance and xBiotech; has received honoraria from Theravance and Green Cross; and has a patent pending in sepsis diagnostics. Other authors have no competing interests.
