# Supplementary material for: Risk stratification biomarkers for Staphylococcus aureus bacteraemia
Source: Clin Transl Immunology. 2020 Feb 13;9(2):e1110. doi: 10.1002/cti2.1110 (PMC7018520; doi:10.1002/cti2.1110)
Supplement: Supplementary file 1 [file CTI2-9-e1110-s001.pdf]

## Supplementary Files

### Supplementary table 1. Biomarker performance in classifying persistent bacteremia, mortality, endovascular infection and infective endocarditis.

Biomarker thresholds to maximize sensitivity and specificity were defined in the previous cohort enriched for persistent bacteremia, mortality, and infective endocarditis [1], and applied to the current study; * IL-6 and sE-selectin thresholds were defined using current cohort data because IL-6 was measured with different assays and sE-selectin sample dilution was different between the two studies. NPV=negative predictive value, PPV=positive predictive value.

| | Biomarker | AUROC (CI) (%) | Threshold (pg/ml) | Sensitivity (%) | Specificity (%) | NPV (%) | PPV (%) |
|---|---|---|---|---|---|---|---|
| 90 day all-cause mortality | IL-8 | 72.5 (60.7 to 84.3) | 80 | 86 | 33 | 93 | 19 |
| | IL-1RN | 62.6 (48.0 to 77.3) | 1507 | 65 | 53 | 93 | 14 |
| | Angpt2 | 59.4 (44.6 to 74.1) | 7147 | 82 | 33 | 92 | 16 |
| | IL-10 | 58.7 (42.6 to 74.8) | 15 | 79 | 40 | 93 | 16 |
| | CCL2 | 58.6 (43.2 to 74.1) | 568 | 75 | 40 | 92 | 14 |
| | IL-6* | 57.0 (41.2 to 72.8) | 130 | 89 | 33 | 93 | 24 |
| | sE-selectin* | 55.5 (43.2 to 67.8) | 46398 | 32 | 93 | 98 | 13 |
| | IL-17A | 52.7 (39.1 to 66.3) | 4 | 61 | 33 | 90 | 8 |
| Attributable mortality | IL-10 | 84.2 (73.1 to 95.3) | 15 | 79 | 83 | 99 | 14 |
| | IL-8 | 81.8 (71.6 to 91.9) | 80 | 85 | 50 | 98 | 12 |
| | IL-1RN | 79.4 (66.0 to 92.8) | 1507 | 65 | 83 | 99 | 8 |
| | IL-6* | 75.2 (53.9 to 96.5) | 130 | 89 | 67 | 99 | 19 |
| | Angpt2 | 72.1 (55.2 to 88.9) | 6200 | 76 | 50 | 98 | 8 |
| | IL-17A | 67.9 (53.2 to 82.5) | 15 | 84 | 17 | 96 | 4 |
| | CCL2 | 61.1 (30.6 to 91.6) | 568 | 75 | 67 | 98 | 10 |
| | sE-selectin* | 51.9 (38.5 to 65.4) | 28940 | 34 | 100 | 100 | 6 |
| Persistent Bacteremia | IL-17A | 73.8 (65.5 to 82.1) | 4 | 50 | 86 | 90 | 40 |
| | IL-10 | 71.3 (62.7 to 79.8) | 11 | 71 | 61 | 82 | 45 |
| | sE-selectin* | 61.9 (52.5 to 71.2) | 36099 | 52 | 73 | 83 | 37 |
| | IL-6* | 55.9 (46.2 to 65.6) | 22 | 29 | 89 | 86 | 33 |
| | Angpt2 | 54.0 (44.5 to 63.6) | 6413 | 26 | 82 | 78 | 30 |
| | CCL2 | 51.6 (41.3 to 61.9) | 560 | 29 | 70 | 71 | 28 |
| | IL-8 | 50.0 (40.5 to 59.5) | 102 | 13 | 91 | 79 | 29 |
| | IL-1RN | 47.6 (37.6 to 57.6) | 1101 | 51 | 52 | 73 | 29 |
| Endovascular infection | IL-10 | 70.8 (62.7 to 78.9) | 4 | 24 | 93 | 76 | 55 |
| | IL-17A | 70.3 (62.2 to 78.4) | 6 | 76 | 54 | 62 | 69 |
| | sE-selectin* | 64.7 (56.2 to 73.3) | 34395 | 60 | 74 | 70 | 65 |
| | IL-1RN | 58.8 (50.0 to 67.7) | 1388 | 64 | 40 | 52 | 52 |
| | Angpt2 | 58.1 (49.2 to 67.0) | 3077 | 50 | 55 | 53 | 52 |
| | CCL2 | 55.3 (46.3 to 64.4) | 662 | 80 | 20 | 50 | 50 |
| | IL-8 | 55.3 (46.3 to 64.3) | 80 | 85 | 18 | 51 | 54 |
| | IL-6* | 49.2 (40.1 to 58.2) | 19 | 23 | 85 | 60 | 52 |
| Infectious endocarditis | IL-17A | 60.2 (47.7 to 72.8) | 6 | 60 | 57 | 62 | 55 |
| | Angpt2 | 56.9 (44.1 to 69.6) | 4446 | 70 | 46 | 60 | 57 |
| | IL-8 | 50.8 (37.8 to 63.9) | 89 | 88 | 19 | 56 | 58 |
| | CCL2 | 50.5 (37.5 to 63.6) | 378 | 44 | 62 | 58 | 49 |
| | IL-10 | 48.5 (35.1 to 61.8) | 8 | 65 | 41 | 56 | 50 |
| | IL-6* | 47.8 (34.8 to 60.9) | 65 | 74 | 35 | 57 | 54 |
| | sE-selectin* | 46.4 (33.4 to 59.5) | 29399 | 91 | 24 | 87 | 30 |
| | IL-1RN | 44.4 (31.3 to 57.5) | 1021 | 47 | 62 | 59 | 50 |

* threshold values calculated on current cohort data

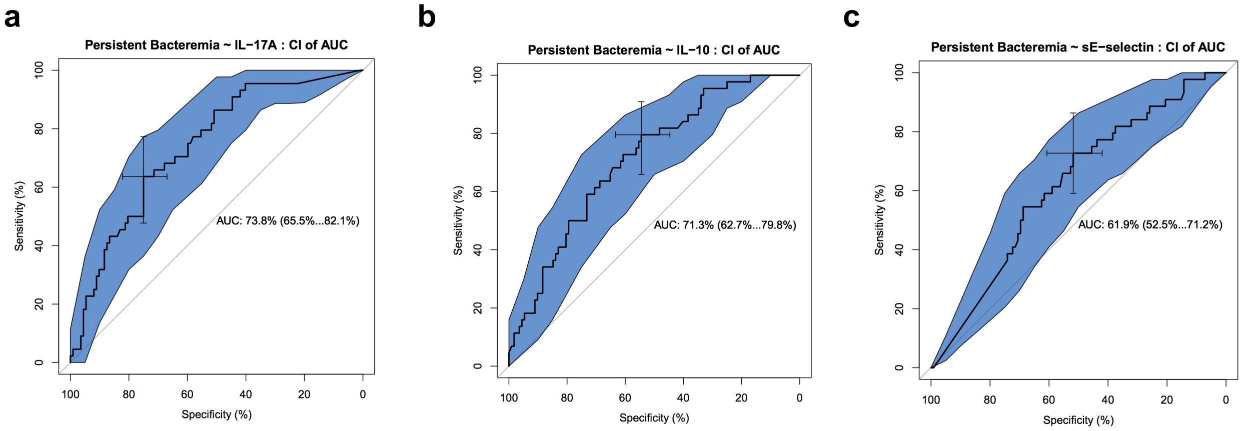


**Supplementary figure 1. (a-c)** Receiver operating characteristic curves of $\log_{10}$ transformed values for IL-17A, IL-10 and sE-selectin for predicting persistent bacteremia. Area under the curves (AUC) are indicated with their 95% confidence interval (CI, shown as blue shading and in parenthesis) and thresholds with maximum combined sensitivity and specificity are indicated, with associated error bars.

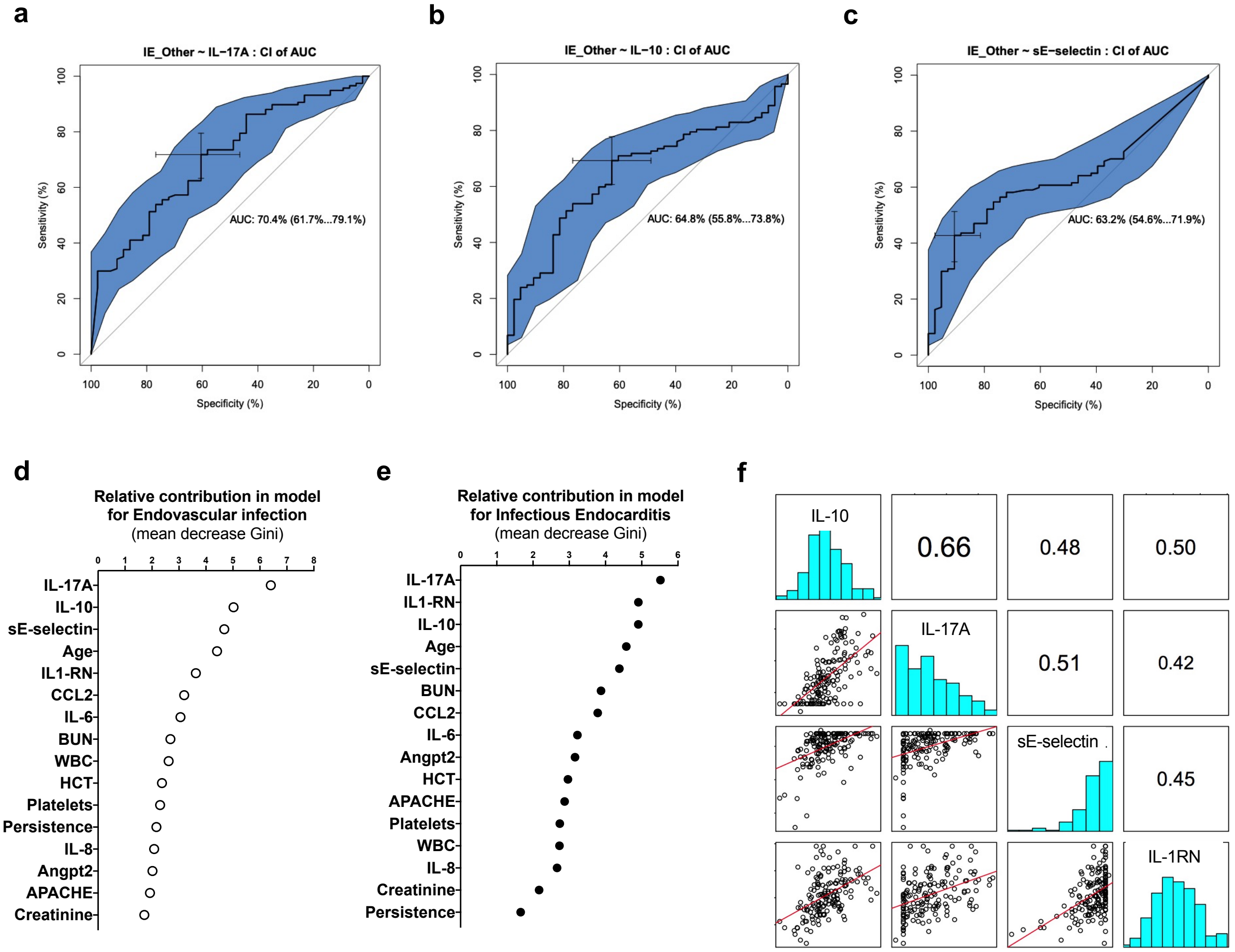


**Supplementary figure 2. (a-c)** Receiver operating characteristic curves of $\log_{10}$ transformed values for IL-17A, IL-10 and sE-selectin for infectious endocarditis (IE) vs all other infection foci. Area under the curves (AUC) are indicated with their 95% confidence interval (CI, shown as blue shading and in parenthesis) and thresholds with maximum combined sensitivity and specificity are indicated, with associated error bars. **(d-e)** Random forest modeling of biomarker classifiers of **(d)** endovascular infection and **(e)** IE. The Mean Decrease Gini of the top 15 most important variables of all biomarkers and clinical risk factors evaluated in the modeling are plotted. **(f)** The top 4 biomarkers with highest contribution to the models for endovascular infection and IE show good Spearman correlations between each other; log transformed patient biomarker values, n=160. Age did not correlate well with any of the above biomarkers (Spearman rho 0.04, 0.02, -0.17 and 0.1 for IL-10, IL-17A, sE-selectin and IL-1RN, respectively).

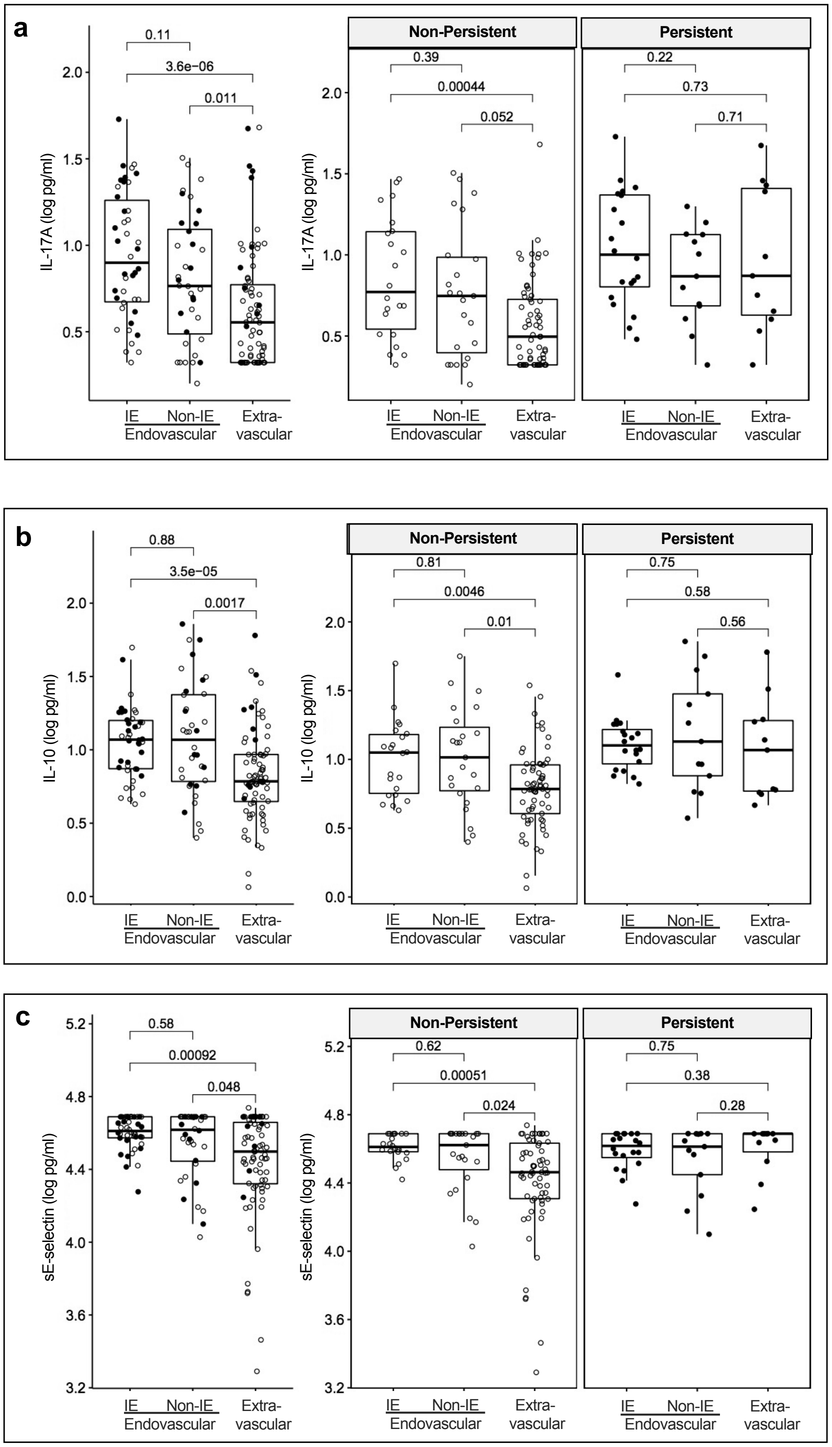


**Supplementary figure 3.** **(a)** IL-17A, **(b)** IL-10, and **(c)** sE-selectin median levels at presentation ($log_2$ scale) are significantly higher in patients with endovascular infections (both infectious endocarditis (IE) and non-IE), when compared to patients with extravascular infections. The association with endovascular infection is observed in subjects without persistent bacteremia (negative blood cultures in < 5 days, open circles). High median biomarker levels were measured in patients with persistent bacteremia (black circles) regardless of diagnosed infection foci. Medians, interquartile ranges (25th and 75th percentiles), and Mann-Whitney *U*-test *P*-values are shown.

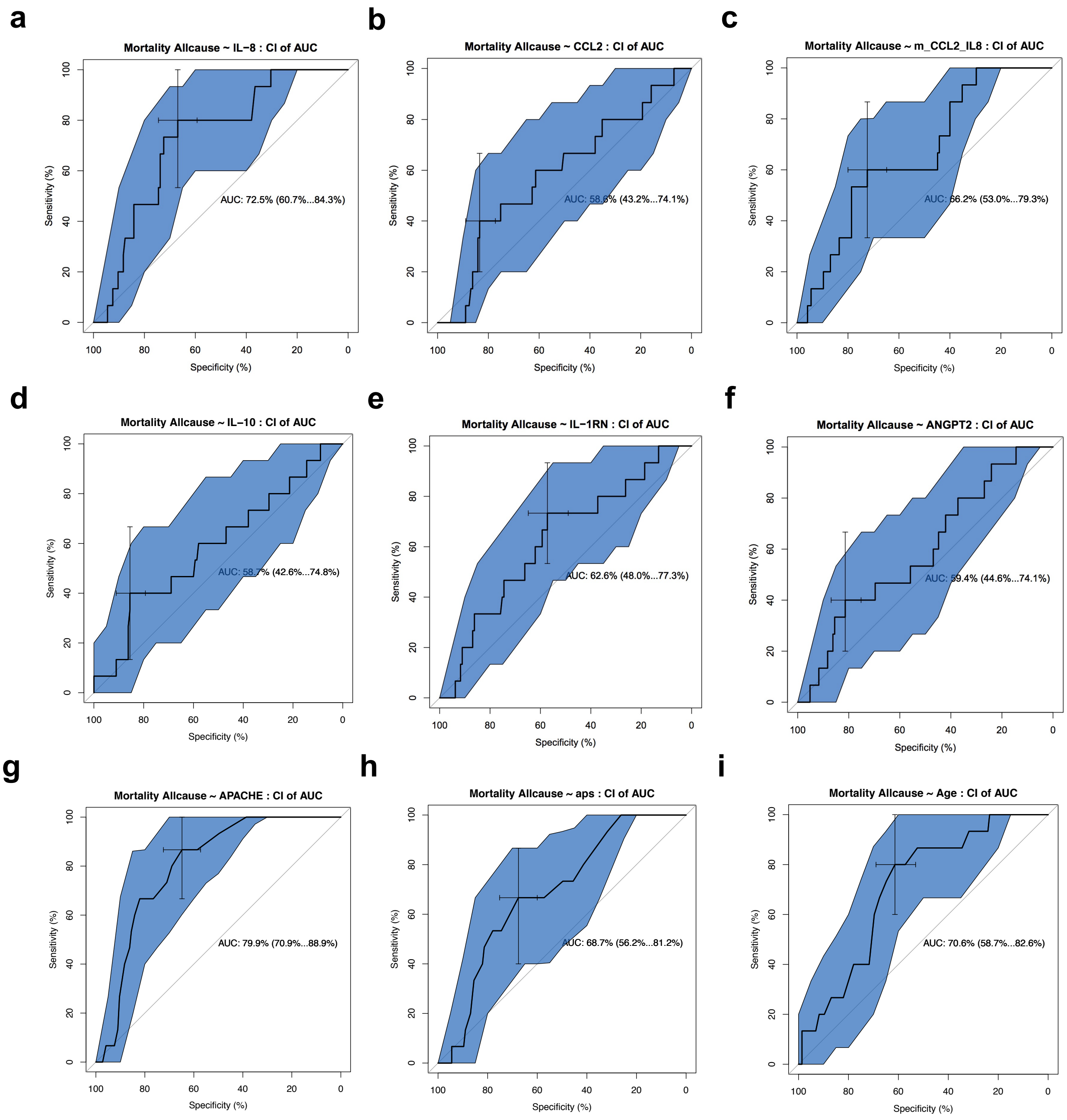


**Supplementary figure 4**. Receiver operating characteristic curves to evaluate the prognostic value for all-cause 90 day mortality of $\log_{10}$ transformed values for **(a)** IL-8, **(c)** CCL2, **(c)** a linear model combining IL-8 and CCL2, **(d)** IL-10, **(e)** IL-1RN, **(f)** Angpt2, **(g)** APACHE II, **(h)** APACHE acute physiology subscore, and **(i)** age. The area under the curves (AUC) are indicated with their 95% confidence interval (CI, shown as blue area and in parentheses) and thresholds with maximum combined sensitivity and specificity are indicated, with associated error bars.

**a**

**30 day mortality**

| Biomarker | AUROC (CI) |
|---|---|
| **IL-1RN** | **0.81** (0.72 to 0.90) |
| **IL-8** | **0.79** (0.70 to 0.87) |
| **APACHE** | **0.76** (0.64 to 0.89) |
| **Angpt2** | **0.67** (0.52 to 0.82) |
| **Age** | **0.67** (0.49 to 0.86) |
| **IL-10** | **0.67** (0.48 to 0.87) |
| **IL-6** | **0.67** (0.49 to 0.84) |
| **CCL2** | **0.59** (0.36 to 0.82) |
| **sE-selectin** | **0.53** (0.41 to 0.65) |
| **IL-17A** | **0.55** (0.38 to 0.72) |

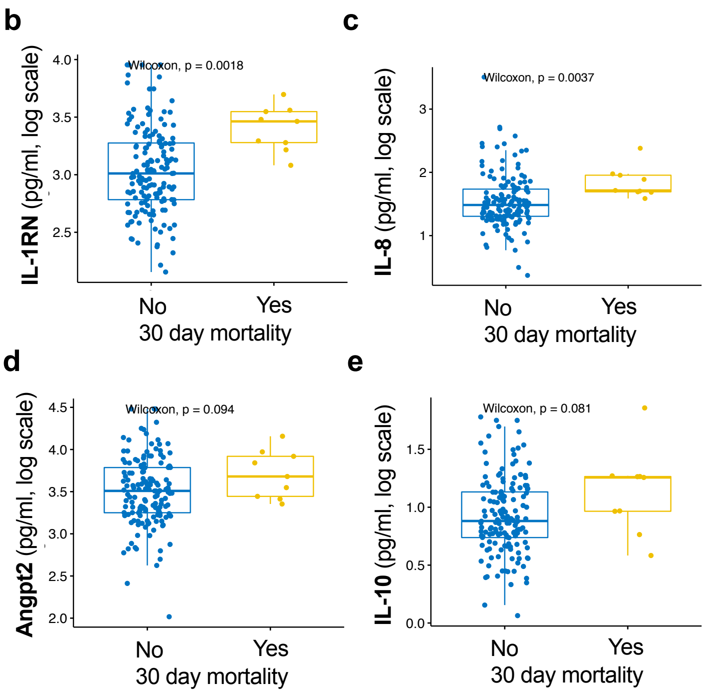


**f**

**In-hospital mortality**

| Biomarker | AUROC (CI) |
|---|---|
| **APACHE** | **0.78** (0.64 to 0.92) |
| **IL-8** | **0.72** (0.57 to 0.87) |
| **IL-1RN** | **0.67** (0.46 to 0.88) |
| **Angpt2** | **0.62** (0.40 to 0.84) |
| **Age** | **0.62** (0.40 to 0.82) |
| **IL-17A** | **0.59** (0.40 to 0.80) |
| **IL-10** | **0.52** (0.25 to 0.78) |
| **CCL2** | **0.50** (0.22 to 0.78) |
| **IL-6** | **0.51** (0.30 to 0.72) |
| **sE-selectin** | **0.51** (0.33 to 0.69) |

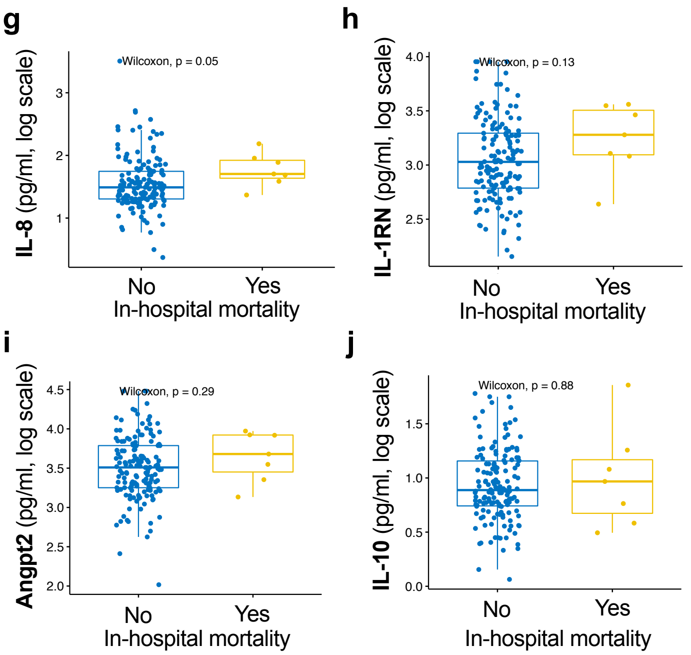


**Supplementary figure 5.** Baseline biomarker area under the receiver operating characteristic curve with 95% confidence intervals (AUROC (CI)) for **(a)** 30 day mortality. Baseline biomarker levels for survivors vs 30 day mortality for **(b)** IL-1RN, **(c)** IL-8, **(d)** Angpt2, and **(e)** IL-10. **(f)** Baseline biomarker AUROC (CI)) for in-hospital mortality. Baseline biomarker levels for survivors vs in-hospital mortality for **(g)** IL-8, **(h)** IL-1RN, **(i)** Angpt2, and **(j)** IL-10. Box and whisker plots showing medians and interquartile range. *P* = unadjusted Mann-Whitney *U*-test (non-parametric Wilcoxon rank sum test). APACHE = APACHE II severity score.

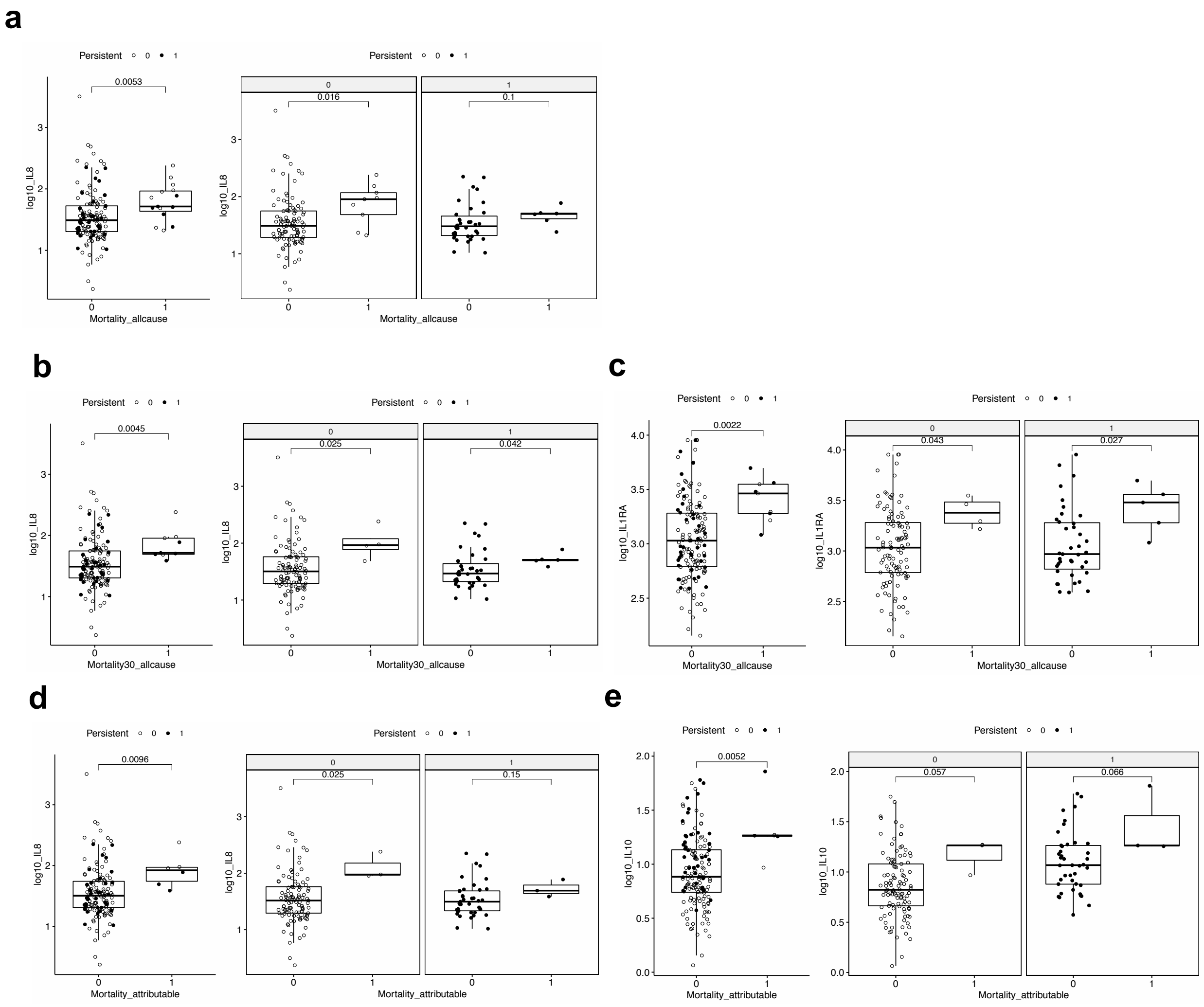


**Supplementary figure 6.** Patients were subset by persistent bacteremia (filled circles) or clearance of positive blood cultures in < 5 days (open circles). The top two biomarkers with *P* < 0.05 (Mann-Whitney *U*-test) in all subjects and the strongest prognostic value for **(a)** 90 day all-cause mortality, **(b-c)** 30 day all-cause mortality, and **(d-e)** attributable mortality are shown. No biomarkers met these criteria for in-hospital mortality. Box and whisker plots show medians and interquartile range. 0 = control (survivor or no persistent bacteremia), 1 = case (fatal outcome or persistent bacteremia).
